# Supercooling of Alaskan Beetle Larvae as a Winter Survival Strategy

**DOI:** 10.1002/smsc.202500058

**Published:** 2025-04-21

**Authors:** Chris J. Benmore, Leighanne C. Gallington, Henry Vu, John G. Duman, Brian M. Barnes, Todd L. Sformo

**Affiliations:** ^1^ X‐Ray Science Division Advanced Photon Source Argonne National Laboratory Lemont IL 60439 USA; ^2^ Department of Biological Sciences University of Notre Dame Box 369 Notre Dame IN 46556 USA; ^3^ Institute of Arctic Biology University of Alaska Fairbanks Fairbanks AK 99723 USA

**Keywords:** cryopreservation, freeze avoidance, glycerol–water, molecular modeling, vitrification, X‐ray diffraction

## Abstract

Insects are able to survive subfreezing temperatures by either limiting ice crystal formation in their bodies or through freeze avoidance. Beetle larvae are able to avoid freezing in winter by dehydrating in the fall months and replacing their body water content with high concentrations of glycerol. This enables the body fluid of the insect to supercool, and even vitrify, recovering unharmed when the temperature warms in the spring. Using nondestructive, high‐energy X‐ray synchrotron diffraction experiments, direct insight into how cryopreservation occurs at the atomic level within the beetle larvae has been obtained. The results shed light on the molecular‐level interactions associated with the mechanism responsible for surviving freezing temperatures. The molecular models of severely dehydrated Alaskan beetle larvae, based on glycerol‐water mixtures, yield a total of 4.2 ± 1.2 intermolecular hydrogen bonds per glycerol molecule at 275 K, in good agreement with existing molecular dynamics simulations. Most importantly, they show that if just over half the body fluid content is water, the water clusters are too small to form ice crystals that cause cellular damage.

## Graphical abstract

Dehydrated Alaskan beetle larvae in Fairbanks, Alaska, adhere to the bark of trees that are embedded in ice in their natural winter microhabitat (bottom). This enables their bodies to deeply supercool and avoid freezing, in contrast to their fully hydrated summer form (top).

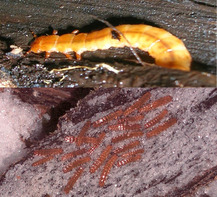

## 1 Introduction

Insects and certain other terrestrial arthropods such as collembola avoid freezing by the use of combinations of concentrations of cryoprotectant polyols such as glycerol, antifreeze proteins, the removal or masking of ice nucleators, and dehydration.^[^ ^1^ ^]^ Some collembola and earthworm cocoons and the Antarctic midge *Belgica antarctica* cryoprotectively undergo extreme water loss by dehydrating until they are in vapor pressure equilibrium with surrounding ice to inhibit freezing.^[^ ^2^ ^]^ Two subspecies of the red flat bark beetle (Coleoptera: Cucujidae), *Cucujus clavipes* *clavipes*, (*C.c. clavipes*, Fabricius), an eastern subspecies, and *Cucujus clavipes puniceus* (*C.c. puniceus*, Mannerheim), a western subspecies, are freeze‐avoiding. Due to differing overwintering habitats, *C.c. clavipes*, from the Great Plains eastward, and *C.c. puniceus*, from interior Alaska, demonstrate differing overwintering capacities in their supercooling ability, glycerol concentration, diapause, and the ability to vitrify into a glassy state.^[^ ^3^ ^]^ *C.c. clavipes* from northern Indiana (IN, 41° 45′ N) show mean winter supercooling points of 250 K and do not dehydrate or diapause in winter.^[3c]^ In contrast, *C.c. puniceus* from Wiseman, Alaska (AK, ≈67° 30′ N) is a seasonal extremophile, having the ability to supercool down to temperatures of 215 K.^[^ ^3d^ ^]^ Interior Alaska has some of the coldest environments in North America, with official recordings showing 221 K in Fairbanks in 1962 and 211 K in 1971 at Prospect Creek Camp 87 km south of Wiseman and ≈107 km south of the northern limit of *C.c. puniceus*.^[^ ^4^ ^]^ Freezing winter temperatures are correlated with loss of body water from summer high levels by a factor of five, concomitant with an increase in hemolymph glycerol concentrations, and the hemolymph displaying thermal hysteresis (in both subspecies) indicative of the presence of antifreeze proteins.^[3b]^ Evidence of limited survival of *C.c. puniceus* from Wiseman, Alaska, under experimental conditions, has been observed down to temperatures of 173 K.^[3d]^ This subspecies has the ability to transition into a glass‐like state at temperatures below 215 K and avoid freezing down to 123 K, adding vitrification as a unique insect overwintering strategy.^[3d]^

Overwintering diapause is exaggerated in insects from arctic and subarctic regions.^[1c,d]^ The main factors contributing to the ability of *C.c. puniceus* to supercool are accumulation of glycerol, production of antifreeze proteins, and extensive dehydration. Antifreeze proteins can mask ice nucleators, inhibit inoculative freezing, and prevent growth of ice embryos, but are largely absent in the bulk fluid of AK and IN beetle larvae, where glycerol is the primary colligative component that decreases the freezing point.^[^ ^3d^ ^]^ Catastrophic cellular damage is a consequence of intracellular ice nucleation.^[^ ^5^ ^]^ However, dehydration and the replacement of water by high concentrations of polyols represent a primary mechanism that enables insects to avoid the formation of ice crystals. A few northern or alpine insects have been shown to be deeply supercool. For example, three freeze‐avoiding Alaska and Canadian Rocky Mountain species that overwinter in willow galls in exposed branches have been shown to supercool down to 210 K.^[^ ^6^ ^]^ Mean temperatures of 219 K have been reported in larvae of the beetle *Pytho deplanatus* that overwinter under the bark of fallen spruce trees in the Canadian Rockies.^[^ ^7^ ^]^

## 2 Building Atomic Models

**Figure 1:**
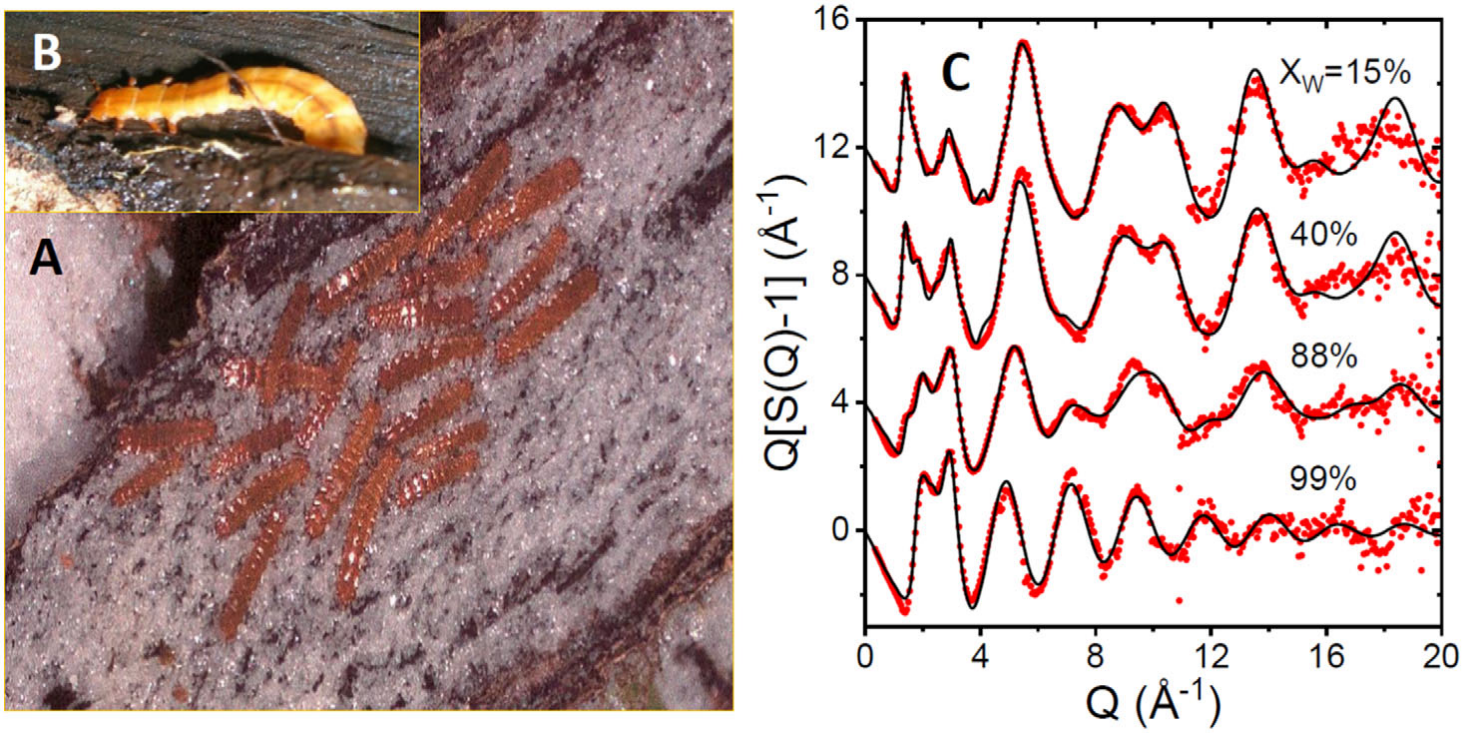
Beetle larvae and their X‐ray diffraction patterns. Alaskan beetle larvae A) under summer conditions and B) under winter conditions attached to ice. C) The X‐ray structure factors measured at 275 K for selected beetle larvae (circles) compared to EPSR models (lines) with different glycerol‐water contents (see Figure S2, Supporting Information for all EPSR fits).

Here we demonstrate an atomic‐level explanation of the mechanism behind the supercooling capacity of AK beetle larvae and have determined the dehydration threshold that AK beetle larvae need to achieve to survive arctic winter temperatures. This has been achieved using high‐energy X‐ray diffraction at an X‐ray synchrotron, and Empirical Potential Structure Refinement (EPSR) simulations to obtain molecular models of the colligative antifreeze glycerol‐water structure in bark beetle larvae of both subspecies (see **Figure** **1**).^[^ ^8^ ^]^ Synchrotron X‐ray diffraction measurements were performed on live AK and IN beetle larvae in transmission geometry, and the recorded intensities were reduced to give the X‐ray structure factor S(*Q*), where momentum transfer (*Q*) is 4*π*sin(*θ*)/*λ*, 2*θ* is the scattering angle, and *λ* is the incident X‐ray wavelength.^[^ ^9^ ^]^ The Fourier transform of S(*Q*) yields the pair distribution functions *g*(*r*), where *ρ* is the atomic number density that describes the probability of finding atom pairs with a given separation r. The peak areas in specific atom–atom *g*(*r*) functions yield coordination numbers, *n*(*r*), that represent the local structural environments in the fluid. Since total X‐ray scattering is a bulk atomic probe, our diffraction patterns are dominated by glycerol and water interactions; this two‐component approximation was used as the basis for our EPSR models.

EPSR is a semi‐rigid molecule Monte Carlo simulation, where all atoms on the molecules are defined using harmonic force constants, with angular and dihedral angles used to describe the molecular geometry and allowed intramolecular rotations (see Figure S1, Supporting Information).^[^ ^8^ ^]^ The algorithm initially uses Lennard‐Jones reference potentials and Coulombic terms to describe the basic intermolecular interactions. As the simulation progresses, an empirical potential is employed to modify these intermolecular interactions to drive the model structure toward the measured S(*Q*) using a Monte Carlo algorithm. Towey et al. have previously used Empirical Potential Structure Refinement (EPSR) to model neutron diffraction data of glycerol–water mixtures.^[^ ^10^ ^]^ In our EPSR modeling of X‐ray diffraction glycerol–water data, we used the partial charges proposed and introduced three rotations around each COH group.^[^ ^10^ ^]^ The X‐ray diffraction data from organic molecules is dominated by the carbon and oxygen atoms, which define the molecular geometry and intermolecular pair correlations in the liquid state. In our analysis, individual beetle larvae were labeled by state and number, for example, AK1 to AK9 and IN2 to IN4. Our results show that *C.c. puniceus* larvae with a mole percent water content of *X*_W_ = 15 mol% H_2_O can avoid freezing and by means of deep supercooling followed by vitrification to a glassy state below Tg ≈ 180 K.^[^ ^11^ ^]^

## 3 Water Clusters

**Figure 2:**
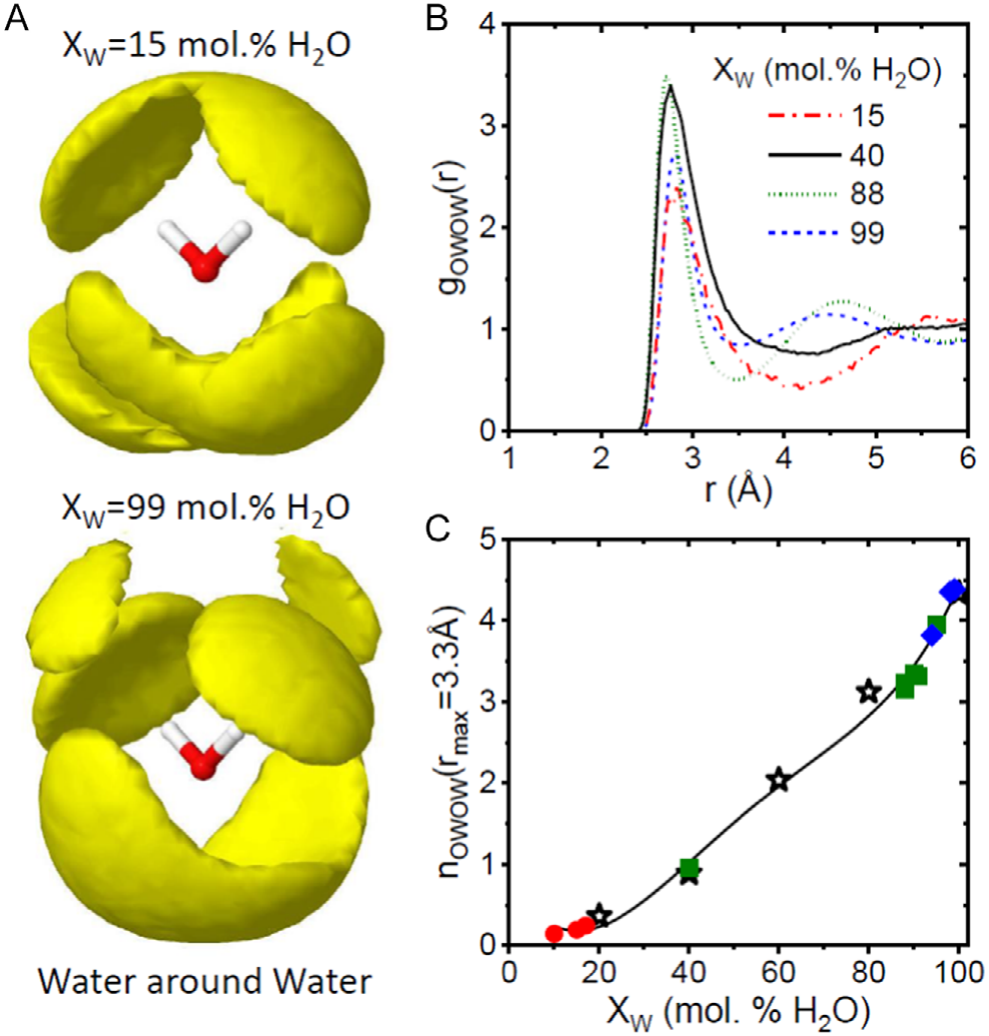
Water coordination environments. A) Spatial distribution functions showing the density of the preferred tetrahedral locations of waters around a central water molecule at low‐ and high‐water concentrations from our EPSR models. B) Oxygen water–oxygen water partial pair distribution functions for selected compositions. C) Corresponding oxygen water–oxygen water (OW–OW) coordination number as a function of water content. Dehydrated (red circles) and hydrated (green squares) Alaskan beetle larvae, and Indiana beetle larvae (blue diamonds) compared to glycerol–water standards (stars).

Water–water partial pair distribution functions are shown in **Figure** **2** and show a near‐constant intermolecular oxygen–oxygen (OW–OW) bond distance of ≈2.8 Å as a function of glycerol‐water content. However, as the amount of water is decreased, the second nearest neighbor peak associated with tetrahedral‐like geometry at ≈4.5 Å moves to longer distances. The corresponding water–water coordination numbers show a steady decrease from ≈4.5 at high water concentrations to almost zero in the most dehydrated beetle larvae models. Water–water spatial density distribution functions for representative *C.c. clavipes* (IN2 *X*_W_ = 99%) and *C.c. puniceus* (AK7 *X*_W_ = 15%) larvae calculated from our 3D models are shown in Figure 2. The *X*_W_ = 99% model spatial distribution function corresponds to a near tetrahedral density distribution normally associated with liquid water. The *X*_W_ = 15% model shows the preferred locations of neighboring water molecules around a central water molecule still try to occupy tetrahedral locations, even in glycerol‐rich environments where there are not enough water molecules alone to achieve this.

**Figure 3:**
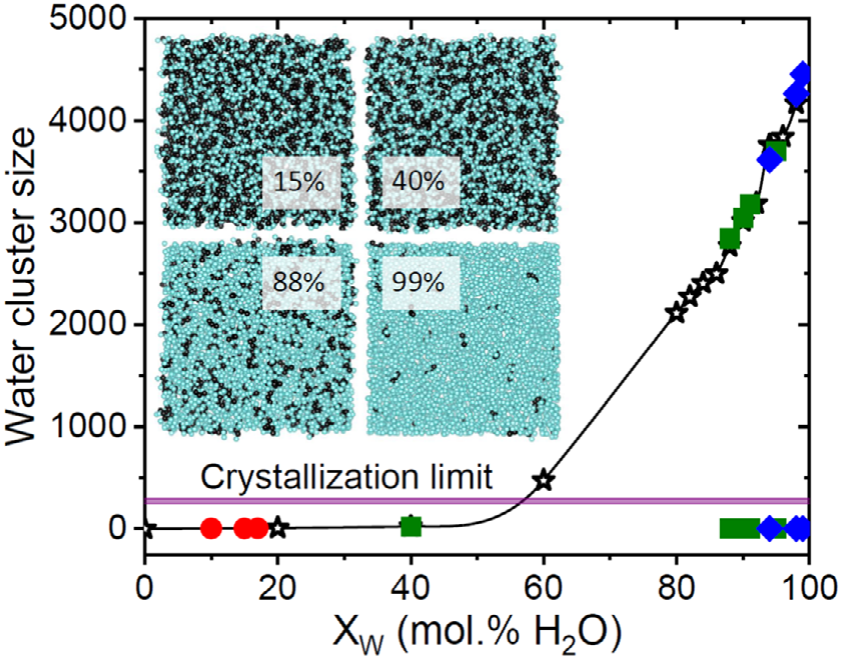
Water cluster size as a function of water content at 275 K. Dehydrated (red circles) and hydrated (green squares) Alaskan beetle larvae and Indiana beetle larvae (blue diamonds) compared to glycerol–water standards (stars). Insets show snapshots of the EPSR models at selected glycerol–water contents, showing small clusters or channels of water oxygen atoms (blue spheres) and glycerol carbon atoms (black spheres) on a subnanometer length scale (hydrogen is omitted for clarity). The crystallization limit of 275 ± 25 water molecules is shown by the magenta band, corresponding to 56 mol% water. The data points at the bottom right correspond to the existence of a few isolated molecules in our simulation box.

Snapshots of the atomistic simulation boxes for selected larvae with different glycerol–water contents are shown in **Figure** **3**. Our glycerol‐rich larvae models show heterogenous glycerol–water mixtures on a subnanometer length scale, and the water‐rich Indiana larvae models show clusters of glycerol–water molecules and channels containing water. This is in agreement with Popov et al. who have argued that at water‐rich concentrations (*X*_W_ > 72 mol% H_2_O), pools of water form, surrounded by a saturated hydrogen‐bonded glycerol–water mixture.^[^ ^11^ ^]^ Microsegregation has also been found in the glycerol–water mixture models in the region *X*_W_ = 50–75 mol% H_2_O, although these were obtained assuming lower densities based on an ideal solution (see Supporting Information).^[10b]^

To investigate this further, a water cluster size distribution analysis was performed on our glycerol‐water and beetle molecular models. The results reveal small water clusters (<25 waters) occur at high glycerol–water concentrations, with larger water clusters (e.g., >275 waters) only occurring above 50 mol% water (although their concentration is very small for <90 mol% water). This result has important implications based on previous infrared studies on water clusters by Pradzynski et al. which found that the minimum number of water molecules needed to form the smallest ice crystals is 275 ± 25 water molecules.^[^ ^12^ ^]^ Consequently, if this dehydration limit is met during the fall months, the AK beetle larvae body fluid will avoid freezing. The crystallization limit, corresponding to a cluster of 275 water molecules, coincides with ≈56 mol% water in larvae body fluid according to our glycerol‐water EPSR models, as determined from our data analysis shown in Figure 3. Bennett et al. have measured the variation in the percentage of body water of beetle larvae in the field throughout the year, which range from lows of ≈28% in winter to highs of ≈64% in summer (see Figure S8, Supporting Information).^[3c]^ The 56 mol% H_2_O threshold for crystallization between the summer hydration and winter dehydration cycle therefore represents a plausible estimation for beetle larvae survival.

## 4 Supercooling and Vitrification

**Figure 4:**
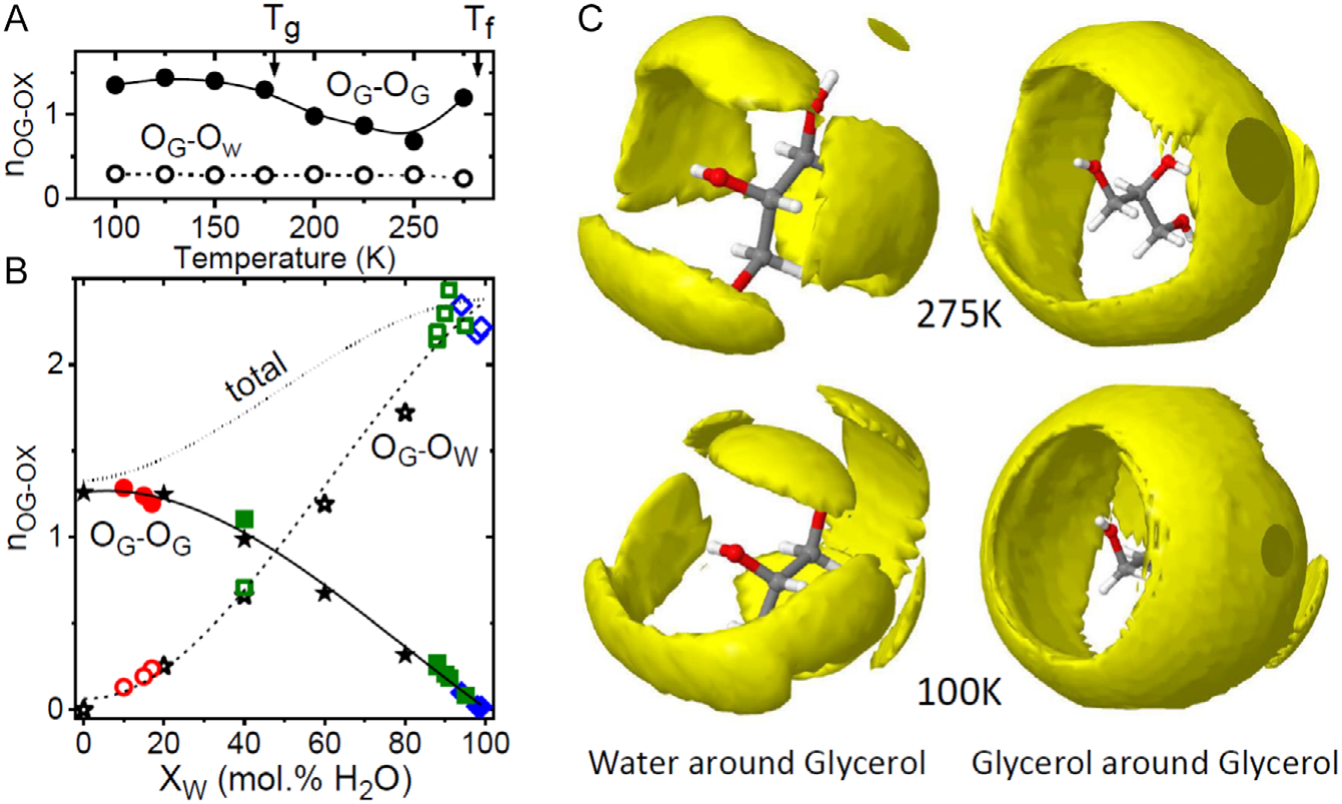
Glycerol coordination environments. The coordination number of glycerol oxygen (OG) or water oxygen (OW) around a central glycerol oxygen atom calculated by integrating out to 3.2 Å A) as a function of (A) temperature and B) water content using the same color code as Figure 3. In (A), the freezing and glass transition temperatures are also marked. In (B), the OG–OG coordination numbers are represented by solid symbols and are fitted with a solid line. The OG–OW coordination numbers are represented by open symbols and are fitted with a dashed line. The total of the solid and dashed lines is represented by the dotted line. C) Spatial distribution functions showing the locations of neighboring molecules around a central glycerol molecule in our EPSR models upon supercooling.

Time‐resolved high‐energy X‐ray diffraction measurements at an incident energy of 100 keV incur negligible radiation damage to biological samples, enabling the in vivo probe of hemolymph supercooling and vitrification of *C.c. puniceus* larvae.^[^ ^13^ ^]^ Consequently, X‐ray data were collected from deeply supercooled and vitreous glycerol‐rich beetle larvae AK7 (*X*_W_ = 15 mol% water) upon cooling and modeled using EPSR. The EPSR structural models show the glycerol oxygen–glycerol oxygen (OG–OG) coordination number exhibits a minimum of around 250 K (illustrated in **Figure** **4**). Our AK7 EPSR model on the beetle larvae based on X‐ray data alone gives a total of 4.2 ± 1.2 intermolecular hydrogen bonds per glycerol molecule at 275 K. This was calculated based on the sum of the OG–OG plus OG–OW coordination numbers since X‐rays are largely insensitive to hydrogen atom locations, and integrating out to a maximum radial distance of 3.2 Å which corresponds to a minimum in the partial pair distribution functions (see Figure S6, Supporting Information). Despite the different hydrogen bond criteria used, this result is in excellent agreement with molecular dynamics (MD) simulations of pure glycerol by Chelli et al. who also find a value of 4.2 hydrogen bonds per glycerol at 278 K.^[^ ^14^ ^]^ The results are however slightly lower (but still within one standard deviation) of those reported by Towey et al. who found 5.7 ± 1.5 hydrogen per glycerol molecule in pure glycerol at 298 K based on EPSR models of neutron diffraction data, but are higher than the MD results on pure glycerol of 2.8 found by Root and Stillinger at 303 K.[^10^, ^15^] The variation in OG–OG and OG–OW coordination is shown in Figure 4, whereby nearest neighbor glycerol oxygen is systematically replaced by water molecules as the water content increases. The crossover point, at which there is equal probability of a glycerol molecule bonding either to another glycerol or water, occurs at *X*_W_ = 48 mol% H_2_O.

Overall, our EPSR models reveal a complex hydrogen‐bonded network structure which is further illustrated in Figure 4 through spatial distribution functions, showing the changes in locations of neighboring molecules around a central glycerol molecule as a function of temperature. Glycerol oxygen is evenly distributed forming a cylinder around the length of the central glycerol molecule. The glycerol–glycerol spatial density map shows a wide variation of possible bonding configurations, consistent with disordered liquid and glassy structures in many different molecular arrangements. This is in contrast to the isolated water molecules, which take up specific bonding and nonbonding locations around a central glycerol molecule. Upon vitrification, the spatial density maps become compressed, consistent with the increased density at low temperatures.^[^ ^16^ ^]^

## 5 Conclusion

Intracellular ice formation is known to be fatal for living organisms that live in extremely cold environments. In this article, we have probed the mechanisms behind ice formation in bark beetle larvae, using a combination of high‐energy synchrotron X‐ray measurements and atomic‐level Monte Carlo modeling using a glycerol‐water model. Based on a water cluster analysis of our structural models, we find that that both homogeneous and heterogenous ice nucleation in the body fluid can be avoided completely in *C.c. puniceus* beetle larvae, if the dehydration remains below a 56 mol% water threshold. In addition, we find that in severely dehydrated Alaskan beetle larvae, there are 4.2 ± 1.2 intermolecular hydrogen bonds per glycerol molecule at 275 K. This result is consistent with previous neutron diffraction studies on the pure glycerol–water system, which have proposed cryoprotective action is manifested through microsegregation that effectively pressurizes and segregates the water to prevent a tetrahedral ice‐like structure from forming, despite the tendency to do so.^[^ ^17^ ^]^ Homogenous nucleation in the bulk fluid due to the ordering of water molecules occurs at much lower temperatures than heterogenous nucleation which arises at interfaces. Small ice clusters are preferentially formed at the surface of antifreeze proteins which mimic the tetrahedral hydrogen bonding patterns of ice, and are able to absorb multiple ice planes despite their low concentrations of <1 wt%.[^5^, ^18^] Indeed, Sformo et al.^[^ ^3d^ ^]^ have suggested the process of overwintering starts in the fall, when antifreeze proteins are produced in addition to the accumulation of glycerol, further preventing ice crystal growth and enhancing the insects’ chances of survival beyond the water‐clustering mechanism described here.

Our methodology could be extended to directly understand the role of cryoprotectants other than glycerol, since high concentrations of glycerol are known to be toxic, and water cluster/ice formation around antifreeze proteins.^[^ ^19^ ^]^ Moreover, it paves the way for the study of more complex systems, to better understand structure–function relationships associated with cryopreservation. For example, vitrification via rapid freezing protocols has been suggested for embryo and organ freezing.^[5a]^ However, this requires high concentrations of a mixture of both permeating (e.g., glycerol) and nonpermeating cryoprotective agents to bypass ice formation. Polyvinylpyrrolidone (PVP) is a nonpermeating cryoprotective agent that possesses inherently lower toxicities and we have previously shown using the combination of high‐energy X‐ray diffraction and EPSR modeling that water preferentially bonds to the PVP polymer chain rather than form water/ice clusters.^[^ ^20^ ^]^

## 6 Experimental Section

6.1

6.1.1

##### High‐Energy X‐ray Diffraction

C*ucujus clavipes* larvae were collected near Fairbanks International Airport in Fairbanks, Alaska (≈64.8° N AK, *C.c. puniceus*), and South Bend, Indiana (IN, *C.c. clavipes*) in January 2017. Larvae were transported to Argonne National Laboratory on ice and subsequently stored at 277 K for 2–3 days (labeled IN water‐rich larvae) or 253 K (labeled hydrated AK larvae) for 4 weeks. A third set of larvae were transported on ice for 2–3 days from Alaska to Argonne and carefully monitored for any signs of water uptake (labeled dehydrated AK larvae). Live larvae were affixed to a metal strip using cyanoacrylate and mounted at beamline 6‐ID‐D of the Advanced Photon Source (see Figure S3, Supporting Information). Larvae were cooled from ambient temperature using an N_2_ cryostream (Oxford Cryosystems) either in 10 K steps (labeled AK1, AK2, IN2) or a ramp rate of 2 K min^−1^ (labeled AK3‐AK9, IN3 and IN4). Water–glycerol standard solutions were prepared to provide controls with a comparable composition to that of larval hemolymph. Water and glycerol were combined in 0:100, 20:80, 40:60, 60:40, 80:20, and 100:0 mole ratios, agitated to form homogenous solutions, and loaded into thin‐walled glass capillaries. Additional water‐rich water–glycerol solutions were prepared with glycerol concentrations between 0 and 20 mol% (2 mol% increments). Typical diffraction patterns were collected using 0.124 Å (100.3 keV) X‐rays on an amorphous silicon‐based area detector (PerkinElmer). Calibration of sample‐detector distance, beam center, tilt rotation, and tilt angle were performed using a CeO_2_ standard. Subtraction of the empty capillary, image summation, and reduction of 2D images to 1D patterns were performed in *GSAS‐II*.^[^ ^21^ ^]^ The larval exoskeleton is mainly comprised of poorly crystallized chitin and characterized by diffuse 110 peaks at ≈1.3 and 1.8–2.0 Å^−1^ and was treated as a background in this analysis and subtracted from the beetle larvae X‐ray patterns. Both the beetle larvae fluid and glycerol‐water data were reduced using standard corrections and normalized using the software *PDFgetX2* to give the X‐ray structure factors, S(*Q*), and corresponding pair distribution functions, G(*r*).^[^ ^22^ ^]^ An important aspect of this work is to create molecular models that reflect the liquid X‐ray diffraction patterns, as such precise *S*(*Q*) and G(*r*) functions are essential. To this end, a recent review of the current software available for accurately analyzing X‐ray diffraction data from liquids has recently been carried out by Gallington et al.^[^ ^23^ ^]^

##### Empirical Potential Structure Refinement (EPSR) Modeling

The intramolecular bond lengths and reference Lennard‐Jones parameters for our EPSR models are given in Table S1 and S2, Supporting Information, respectively. The EPSR models contained ≈14 000 atoms, corresponding to cubic box sizes of *V* ≈ 50 × 50 × 50 Å^3^, and were averaged over 2000 configurations. Details of the simulation box parameters are given in Table S3, Supporting Information. The atomic number density of Behrands et al. was used in our EPSR models, which differed from previous studies which assumed an ideal mixture of water and glycerol (see Figure S5A, Supporting Information).[^10^, ^24^] Using lower densities consistent with an ideal solution led to EPSR models which contained unphysically large voids in the liquids when forced to fit the X‐ray S(*Q*). The densities reported by Behrands et al. yielded good fits to the S(*Q*)'s across the entire compositional range with no voids in the simulation boxes.^[^ ^24^ ^]^ The glycerol–water mixtures showed major systematic changes in the relative height of the glycerol intramolecular C1–C2 glycerol distance in the total pair distribution function T(*r*) = 4*πrr*G(*r*) (see Figure S4 and S5C, Supporting Information), which was subsequently used as a calibration curve for the larvae data. Essentially the same calibration curves were found for the second intramolecular C1‐C3 glycerol distance and third nearest neighbor peak in T(*r*) corresponding to the oxygen water–oxygen water (OW–OW) distance confirming the efficacy of the method (see Figure S5B,D, Supporting Information). Consequently, the following methodology was used; the beetle larvae data were initially analyzed using *PDFgetX2* assuming an approximate glycerol‐water mixture based on the shape of the S(*Q*). The calibration curve in Figure S5D, Supporting Information, was used to determine the glycerol–water ratio, that is, the mole percent water content *X*_W_, and the diffraction data was reanalyzed with that composition to obtain a revised S(*Q*). This process was repeated iteratively until the composition and calibration curve were consistent. An EPSR model of glycerol–water was then constructed based on the local structure calibration and fit to the beetle larvae X‐ray S(*Q*). The extracted water–water coordination species as a function of water content is shown in Figure S7, Supporting Information. Lastly, the criteria of whether a molecule is in a cluster is determined by the separation of specified pairs of atoms. A water cluster is determined from the oxygen atoms in H_2_O molecules. Any two water molecules whose oxygen atoms are determined to be at a distance of 3.5 Å of each other are said to be in the same cluster. All the molecules within the simulation box which are also in the same cluster are tallied and used to generate a distribution of cluster size, where the size is the number of molecules in the cluster.

##### Statistical Analysis

Statistical errors on the X‐ray data were determined based on a Poisson distribution and found to lie within the scatter of the data points. Long wavelength systematic errors in the extracted X‐ray S(*Q*), arising from effects such as variations in the *Q*‐dependent attenuation factors or asymmetrical electron cloud distributions, were removed by setting G(*r* < 1.2 Å) = 0.

## Conflict of Interest

The authors declare no conflict of interest.

## Author Contributions

**Chris J. Benmore**: conceptualization (lead); data curation (equal); formal analysis (lead); investigation (equal); methodology (lead); validation (lead); visualization (lead); writing—original draft (lead); writing—review & editing (supporting). **Leighanne C. Gallington**: data curation (equal); formal analysis (equal); investigation (supporting); methodology (supporting); writing—original draft (equal); writing—review & editing (equal). **Henry Vu**: investigation (supporting); resources (supporting); writing—review & editing (equal). **John G. Duman**: investigation (supporting); resources (supporting); writing—review & editing (equal). **Brian M. Barnes**: investigation (supporting); resources (supporting); writing—review & editing (equal). **Todd L. Sformo**: conceptualization (supporting); data curation (supporting); investigation (supporting); resources (lead); validation (supporting); writing—original draft (equal); writing—review & editing (equal).

## Supporting information

Supplementary Material

## Data Availability

The data that support the findings of this study are available from the corresponding author upon reasonable request.
