# Supplementary material for: Supercooling of Alaskan Beetle Larvae as a Winter Survival Strategy
Source: Small Sci. 2025 Apr 21;5(6):2500058. doi: 10.1002/smsc.202500058 (PMC12168606; doi:10.1002/smsc.202500058)
Supplement: Supplementary file 1 — Supplementary Material [file SMSC-5-2500058-s001.pdf]

Supporting Information

Supercooling of Alaskan beetle larvae as a winter survival strategy.

Chris J. Benmore, Leighanne C. Gallington, Henry Vu, John G. Duman, Brian M. Barnes and Todd L. Sformo*

Supporting information tables

Intramolecular bond	Bond length (Å)
**OW-HW**	0.965
**OG-CG, OG-CC**	1.45
**OG-HO**	0.97
**CG-CC**	1.54
**HG-CC, HG-CG**	1.08

Table S1. Glycerol intramolecular bond lengths.

Atom	ε (kJ mol^{-1})	σ (Å)	q (e)
**OW**	0.65	3.166	-0.82
**HW**	0.0	0.0	+0.41
**OG**	0.65	3.1	-0.624
**CG**	0.80	3.7	+0.107
**CC**	0.80	3.7	+0.170
**HO**	0.0	0.0	0.392
**HG**	0.0	0.0	0.063

Table S2. EPSR reference potential parameters.

Beetle	AK1	AK2	AK3	AK4	AK5	AK6
**Atomic number Density (Å^{-3})**	0.11648	0.10884	0.10884	0.10780	0.10448	0.10715
**Number of waters**	580	2860	2860	3060	3705	3185
**Number of Glycerols**	870	390	390	340	195	315
**Beetle**	**AK7**	**AK8**	**AK9**	**IN2**	**IN3**	**IN4**
**Atomic number Density (Å^{-3})**	0.11621	0.11603	0.11632	0.10522	0.10178	0.10088
**Number of waters**	196	109	171	3619	4263	4455

**Number of Glycerols**	955	981	969	231	87	45

Table S3. EPSR simulation box parameters.

Supporting Information figures

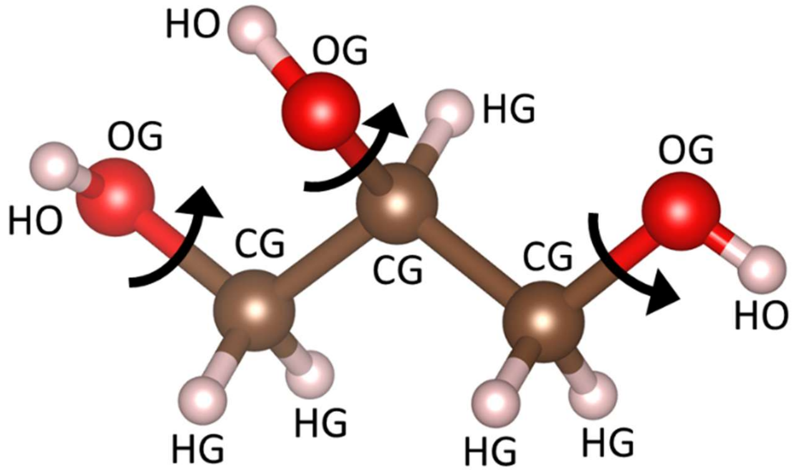

Figure S1. Glycerol molecule atom labels and allowed rotations.

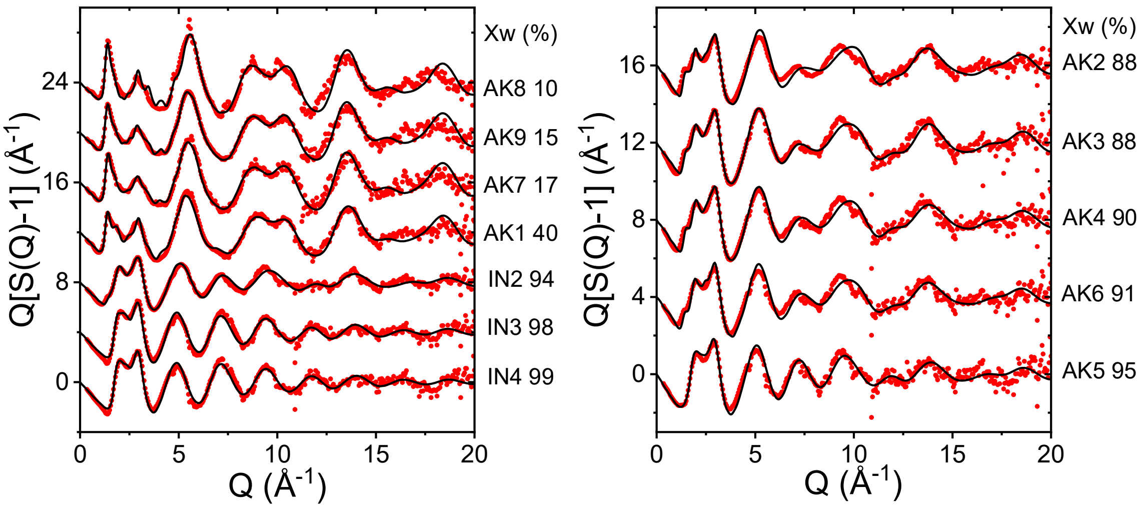

Figure S2. EPSR x-ray glycerol-water fits (lines) of all measured AK and IN beetle larvae (red circles) measured at 275K. The mole percent water content X_W for each is given in the sidebar.

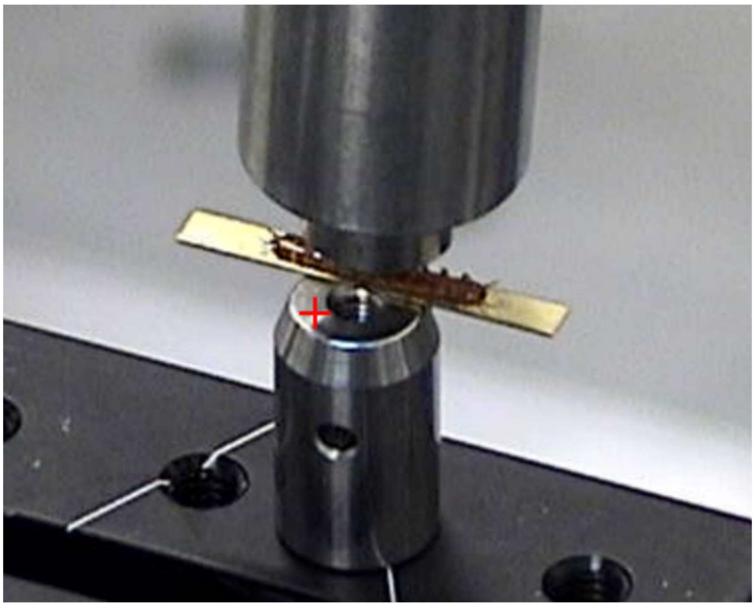

Figure S3. Experimental setup with the beetle larvae mounted on a brass plate, perpendicular to the x-ray beam and the cryostream above.

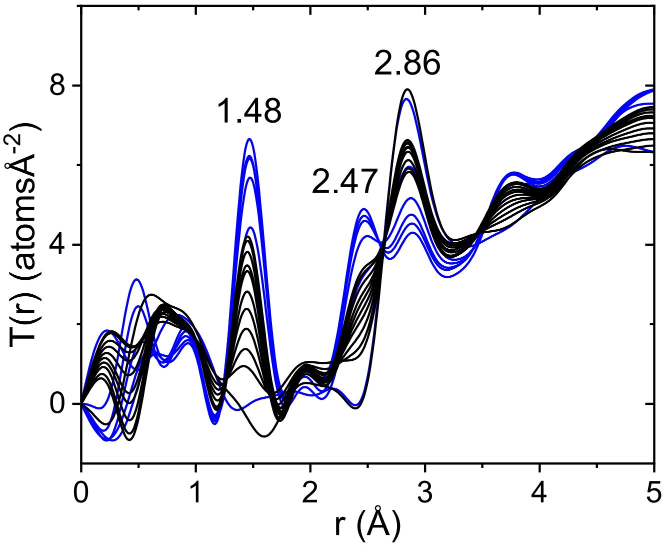

Figure S4. The glycerol-water total pair distribution functions showing the nearest neighbor intra-molecular C-C bonds and OW-OW water-water peak. Water:glycerol mixtures in 0:100, 20:80, 40:60, 60:40, 80:20, and 100:0 mol.% ratios are shown in blue. Water:glycerol solutions between 0 and 20 mol% glycerol in 2 mol% increments are shown in black.

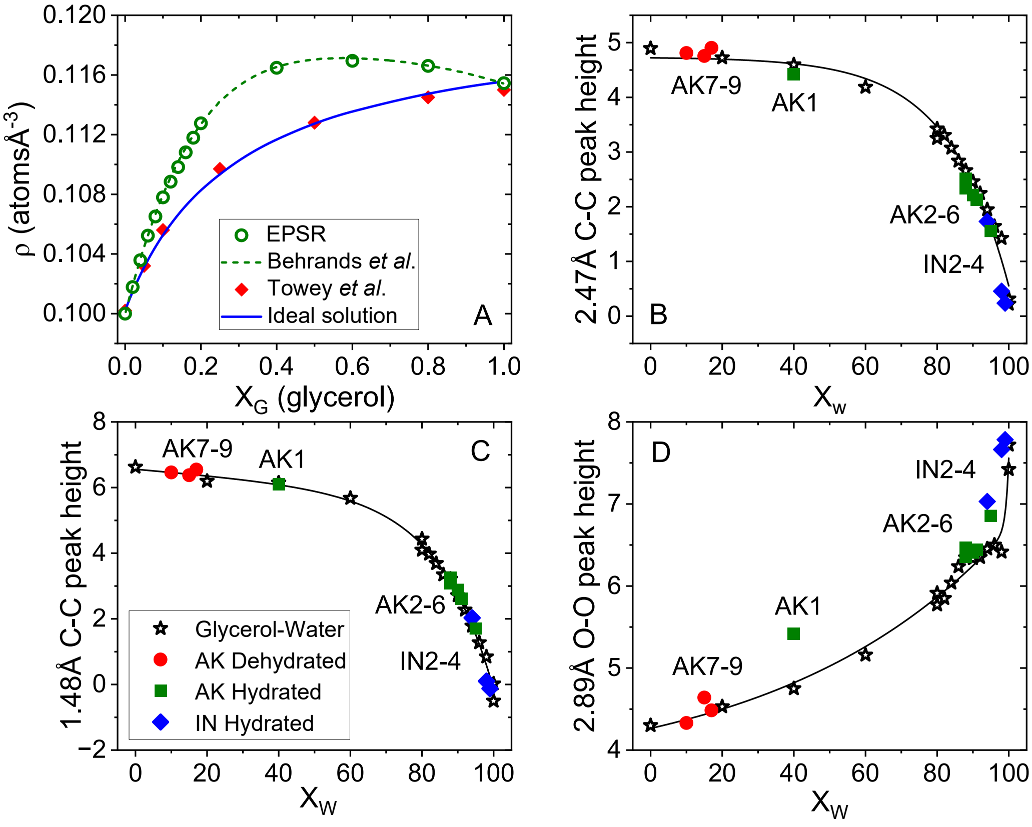

Figure S5. (A) Atomic number densities of measurements of Behrands *et al*. (solid line) and EPSR models (circles) compared to an ideal solution (dashed line). Calibration curves for the nearest neighbor peak heights corresponding to the (B) 1st intramolecular C-C bond (C) 2nd C-C intramolecular distance and (D) O-O intermolecular water distance in the x-ray pair distribution function. Solid symbols represent the

beetle larvae measurements compared to the glycerol-water mixture (stars) as a function of water content.

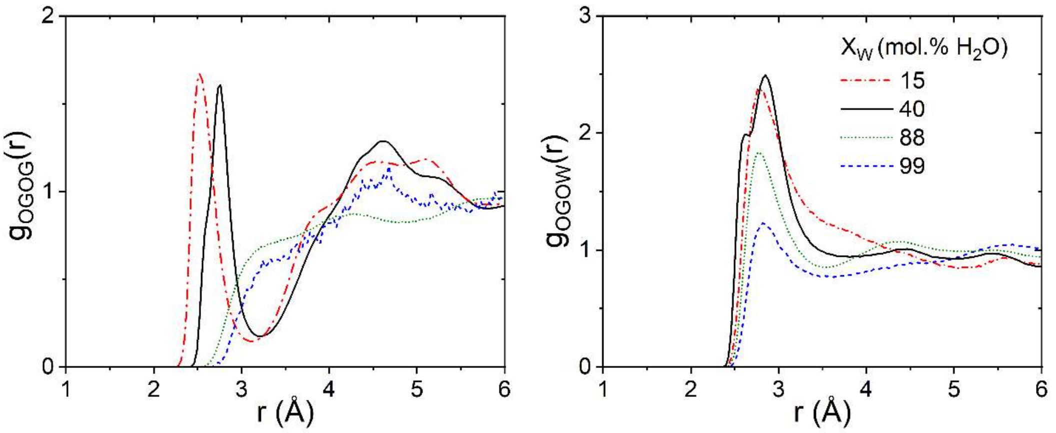

Figure S6. Oxygen glycerol-oxygen glycerol (OG–OG) and oxygen glycerol-oxygen water (OG–OW) partial pair distribution functions for selected compositions.

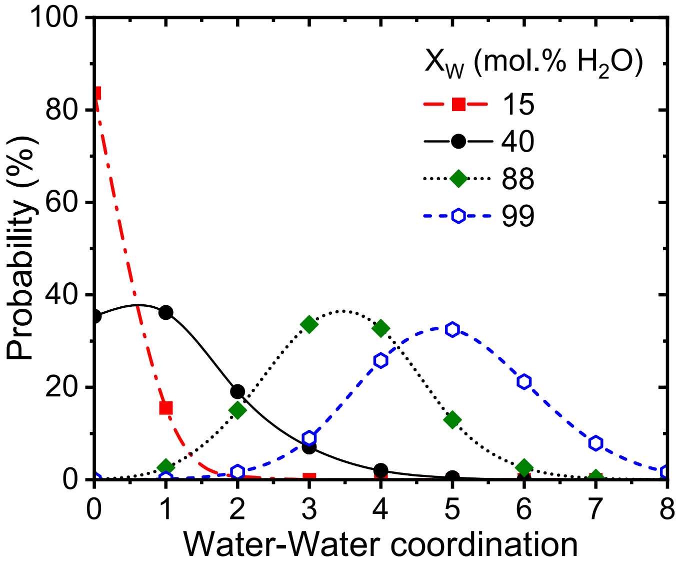

Figure S7. Water-water coordination species as a function of water content.

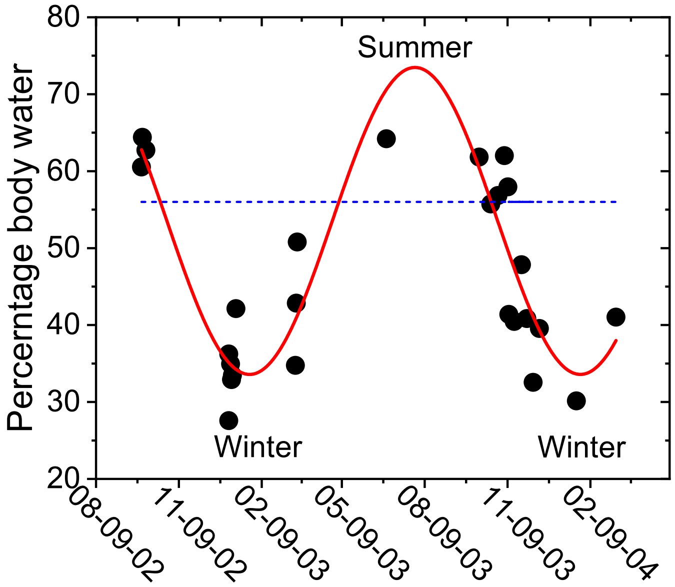

Figure S8. Percent body water in beetle larvae measured in the field by Sformo *et al*. over a period of two winters in Fairbanks Alaska (circles) fit with a sinusoidal curve (red line). The dashed blue line represents the 56 mol.% threshold below which small water clusters in glycerol water do not crystallize.
